# Influence of the growth pattern on cortical bone thickness and mini-implant stability

**DOI:** 10.1590/2177-6709.25.6.033-042.oar

**Published:** 2020

**Authors:** Carolina Carmo de Menezes, Sérgio Estelita Barros, Diego Luiz Tonello, Aron Aliaga-Del Castillo, Daniela Garib, Silvio Augusto Bellini-Pereira, Guilherme Janson

**Affiliations:** 1 Universidade de São Paulo, Faculdade de Odontologia de Bauru, Departamento de Ortodontia (Bauru/SP, Brazil).; 2 Universidade Federal do Rio Grande do Sul, Faculdade de Odontologia, Departamento de Ortodontia (Porto Alegre/RS, Brazil).

**Keywords:** Orthodontics, Skeletal anchorage, Stability

## Abstract

**Introduction:**

Controversial reports suggest a relationship between growth pattern and cortical alveolar bone thickness, and its effect in the use of mini-implants.

**Objective:**

The main purpose of this study was to assess the influence of the growth pattern on the cortical alveolar bone thickness and on the stability and success rate of mini-implants.

**Methods:**

Fifty-six mini-implants were inserted in the buccal region of the maxilla of 30 patients. These patients were allocated into two groups, based on their growth pattern (horizontal group [HG] and vertical group [VG]). Cortical thickness was measured using Cone Beam Computed Tomography. Stability of mini-implants, soft tissue in the insertion site, sensitivity during loading and plaque around the mini-implants were evaluated once a month. Intergroup comparisons were performed using *t* tests, Mann-Whitney tests, and Fisher exact tests. Correlations were evaluated with Pearson’s correlation coefficient.

**Results:**

The cortical bone thickness was significantly greater in the HG at the maxillary labial anterior region and at the mandibular buccal posterior and labial anterior regions. There was a significant negative correlation between Frankfort-mandibular plane angle (FMA) and the labial cortical thickness of the maxilla, and with the labial and lingual cortical bone thicknesses of the mandible. No significant intergroup difference was found for mini-implant mobility and success rate. No associated factor influenced stability of the mini-implants.

**Conclusions:**

Growth pattern affects the alveolar bone cortical thickness in specific areas of the maxilla and mandible, with horizontal patients presenting greater cortical bone thickness. However, this fact may have no influence on the stability and success rate of mini-implants in the maxillary buccal posterior region.

## RESUMO

**Introdução:**

Relatos controversos sugerem uma relação entre o padrão de crescimento e a espessura do osso alveolar cortical e seu efeito no uso de mini-implantes.

**Objetivo:**

Avaliar a influência do padrão de crescimento na espessura do osso cortical alveolar e na estabilidade e taxa de sucesso dos mini-implantes.

**Métodos:**

Cinquenta e seis mini-implantes foram inseridos na região vestibular da maxila de trinta pacientes. Esses pacientes foram alocados em dois grupos, com base em seu padrão de crescimento (grupo horizontal [GH] e grupo vertical [GV]). As espessuras corticais foram medidas por meio de tomografias computadorizadas de feixe cônico. A estabilidade dos mini-implantes, tecido mole no local de inserção, sensibilidade durante a carga e o acúmulo de placa ao redor dos mini-implantes foram avaliados uma vez por mês. As comparações intergrupos foram realizadas por testes *t* de Student, testes de Mann-Whitney e testes exatos de Fisher. As correlações foram avaliadas com o coeficiente de correlação de Pearson.

**Resultados:**

A espessura do osso cortical foi significativamente maior no GH na região vestibular anterior da maxila e nas regiões vestibulares posterior e anterior da mandíbula. Houve uma correlação negativa significativa entre o ângulo do plano mandibular (FMA) e a espessura da cortical vestibular da maxila, e com a espessura das corticais vestibular e lingual da mandíbula. Nenhuma diferença significativa entre os grupos foi encontrada com relação à mobilidade do mini-implante e taxa de sucesso. Nenhum fator associado influenciou a estabilidade dos mini-implantes.

**Conclusões:**

O padrão de crescimento afeta a espessura da cortical óssea alveolar em áreas específicas da maxila e mandíbula, com pacientes horizontais apresentando maior espessura da cortical óssea. No entanto, esse fato pode não ter influência na estabilidade e na taxa de sucesso dos mini-implantes na região vestibular posterior da maxila.

## INTRODUCTION

The use of mini-implants as anchorage has become a common routine due to its high predictability and practicality.^1^ ^,^ ^2^ Stability of these anchorage devices is related to several factors, such as: site of insertion,^1^ oral hygiene,^3^ bone quality^4^ and mostly the primary stability and load intensity.^5^

Cortical bone thickness is considered a determinant factor for primary stability of mini-implants. It is suggested that greater thickness of the alveolar cortical bone is associated with greater chances of primary stability and, consequently, better success rate.^4^ ^-^ ^6^ In addition, associations between cortical bone thickness and vertical growth pattern have been evidenced, and the majority of the studies show that subjects with vertical growth pattern present thinner cortical bone, when compared with subjects with normal or horizontal growth.^7^ ^-^ ^11^

These associations could lead to the speculation that the vertical growth pattern could have some influence on the stability and success rate of mini-implants. However, only few studies have evaluated this direct association and the findings are controversial.^1^ ^,^ ^2^ ^,^ ^12^

Miyawaki el al.^12^ reported that a higher mandibular plane angle is associated with greater failure of mini-implants. Contrarily, other studies^1^ ^,^ ^2^ reported that there is no correlation between the mandibular plane angle and mini-implants success rate. Recent evidence supports the assumption that high-angle patients present narrower inter-radicular cortical bone thickness, when compared to low-angle patients, and this fact may play a role in mini-implants success.^10^ Therefore, more studies are needed to confirm this association.

Based on this controversy and because cortical bone thickness could depend on the growth pattern, and it is considered an important factor related to mini-implant stability, it should be more deeply studied. For this reason, the primary objective of this study was to evaluate the influence of the vertical growth pattern on the alveolar bone cortical thickness and secondarily, assess the factors related to stability and success rate of orthodontic mini-implants.

## MATERIALS AND METHODS

This study was approved by the Ethics in Research Committee of *Universidade de São Paulo, Faculdade de Odontologia de Bauru* (protocol #069/2009). A minimum sample size of 14 participants per group was necessary to provide 80% of test power, at a significance level of 0.05, to detect an intergroup difference of 0.5 mm in the alveolar bone cortical thickness, with a previously reported standard deviation of 0.45.^13^

The sample comprised 30 patients divided into two groups, according to their growth pattern, based on the Frankfort mandibular plane angle (FMA): FMA values smaller than the sample mean (24.35°) indicated horizontal growth pattern, and FMA values greater than 24.35° showed vertical growth pattern.^14^ Therefore, the horizontal group (HG) comprised 15 patients that overall had 26 mini-implants (MI) and the vertical group (VG) consisted of 15 patients that overall had 30 MI. 

The inclusion criteria consisted on: Class I and Class II malocclusion patients, presence of complete permanent dentition, need of at least one premolar extraction in the maxilla, and cases in which skeletal anchorage was required for anterior retraction in order to prevent any anchorage loss, such as severe Class I biprotrusion and severe Class II cases. 

The exclusion criteria involved patients with mini-implants used for other biomechanics need, and the presence of any local or systemic condition that could influence stability of the mini-implants, as active periodontal disease, smoking and diabetes.

A total of 56 self-drilling mini-implants were evaluated. Thirty-eight mini-implants (1.5-mm diameter, 7-mm length) were inserted by one orthodontist (SEB) following the surgical technique that uses a coaxial radiographic positioner associated with a three-dimensional radiographic-surgical guide.^15^ The other 18 mini-implants (1.6-mm diameter, 8-mm length) were inserted by another orthodontist (CCM) following the guide-free technique, based on tooth crown references.^16^ Both operators were previously calibrated. All MI were inserted into the buccal, posterior maxillary region (38 between second premolars and first molars, 3 between first molars and second molars, and 15 between first premolars and second premolars). Immediate loading (100-250 g) was applied to all mini-implants using elastic chains. Information regarding the insertion techniques, site of insertion, and mini-implant characteristics used in the study are shown in Table 1.

**Table 1:** Information regarding the insertion techniques, site of insertion, and mini-implant characteristics used in the study.

Operator	Insertion technique	Sample		Site of insertion	Mini-implant characteristics
		n	%		
SEB	Three-dimensional radiographic-surgical guide^15^	38	64.86	All - between second premolars and first molars	1.5-mm diameter, 7-mm length
CCM	Guide-free technique^16^	18	32.14	(n=3) - between first molars and second molars (n=15) - between first premolars and second premolars	1.6-mm diameter, 8-mm length

Lateral cephalograms were evaluated to determine the vertical growth pattern (FMA). The cephalometric tracings and landmark identifications were performed on acetate paper by one investigator (CCM) and then digitized with Numonics Accugrid XNT digitizer (Houston Instruments, Austin, TX). These data were then stored in a computer and analyzed with Dentofacial Planner (version 7.0; Dentofacial Software, Toronto, Ontario, Canada). 

**Figure 1:**
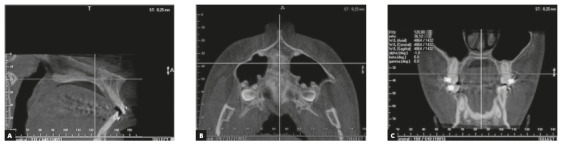
Bispinal reference line, to standardize the sagittal and axial sections (A and B). Reference line between the buccal bone crests of maxillary first molars (C).

Cone Beam Computed Tomography (CBCT) images were obtained using the 3D i-CAT cone beam system (Imaging Sciences International, Hatfield, PA) using a protocol of 120 kV, 36.12 mA, 8 cm field of view and voxel size of 0.25 mm. CBCT images were analyzed using an i-CAT Viewer software (XoranCat-Xoran Technologies, Ann Arbor, MI). The CBCT scans were obtained during the alignment and leveling phase and before mini-implant insertions. The reference chosen to standardize the axial and sagittal plane was the bispinal line, coinciding with the vertical and horizontal planes, respectively (Figs 1A and B). The reference used to standardize the coronal plane was a line between the buccal bone crests of the maxillary first molars^17^ (Fig 1C).

**Figure 2:**
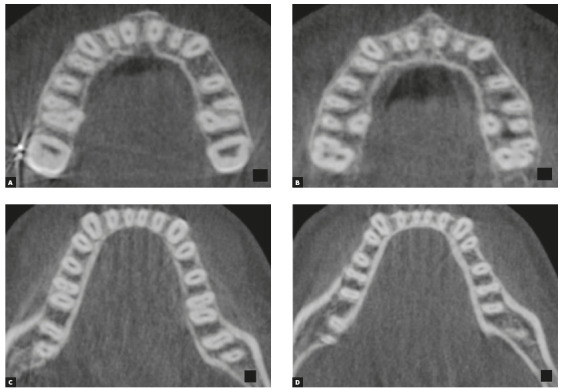
Axial sections of the maxilla at 3.0 and 6.0 mm apical to the cemento-enamel junction of the right maxillary first molar, respectively (A and B). Axial sections of the mandible at 4.0 mm and 8.0 mm apical to the cemento-enamel junction of the right mandibular first molar, respectively (C and D).

After obtaining the maxillary and mandibular standardized axial sections,^17^ two axial slices were selected passing 3.0 mm and 6.0 mm (Fig 2A, 2B); 4.0 mm and 8.0 mm (Fig 2C, 2D) apical to the cement-enamel junction, for the maxilla and the mandible, respectively.^17^

**Figure 3:**
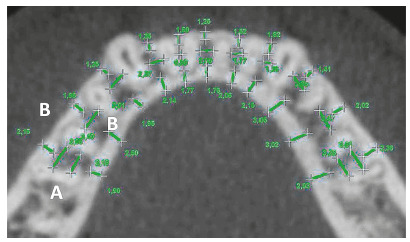
A) Interradicular distance (mesiodistal dimension). B) Thickness of the buccal and lingual cortical bone in the center of the interradicular septum.

Measurements of the buccal and lingual cortical bone thickness were performed once on the CBCT scans, by an adaptation of the method advocated by Lee et al.^18^ Initially, the interradicular distance was measured for each tooth. This distance was measured parallel to the arch contour, connecting the mean portion of each root, and defined as the smallest distance between the radicular surface of the adjacent teeth (Fig 3A). These measurements served as a guide for the subsequent measurements.^18^ Thickness of the alveolar cortical bone was measured, for each tooth, from its outermost portion, perpendicular to the arch form, to its most inner portion, in the center of the interradicular spaces on the buccal and lingual sides (Fig 3B).

The cortical bone thickness measurements were grouped and the averages of the following regions were calculated for each patient and used in the statistical analyses:

» Maxillary buccal posterior region thickness (MxBP).
» Maxillary labial anterior region thickness (MxLA).
» Maxillary palatal posterior region thickness (MxPP).
» Maxillary palatal anterior region thickness (MxPA).
» Mandibular buccal posterior region thickness (MdBP).
» Mandibular labial anterior region thickness (MdLA).
» Mandibular lingual posterior region thickness (MdLP).
» Mandibular lingual anterior region thickness (MdLgA).

Mini-implant stability was assessed by monthly measurements from the time of insertion (primary stability) until its removal. The horizontal amount of mobility was linearly measured (mm). The mean observation period was 9.62 and 1.67 months, for the success and failure groups, respectively. This measurement was performed with the aid of an adjustable telescopic rod (ATR).^19^ The ATR was capable of connecting with the mini-implant head and was associated with an orthodontic tension gauge (Correx series 040-712-00, Dentaurum Orthodontics) which applied a force of 400g.^19^ Thus, using reference points, the ATR length was adjusted according to the distance between the mini-implant head and the chosen point. Then, distances could be measured, before and after force application, with a digital caliper (Mitutoyo 500-144B, Mitutoyo, Japan). After the force was applied, if the distance measured by the caliper was similar before and after force application, the mini-implant was considered stable. This method was described in detail and validated in a previous study.^19^ The mean of monthly measurements was used for statistical analysis. The success rate was defined as the number of mini-implants that remained clinically stable, to ensure the orthodontic load during the overall observation period, divided by the total number of evaluated mini-implants.

The factors that could interfere with mini-implant stability were clinically evaluated. Three insertion sites (IS) were considered: attached gingiva; mucogingival line; and alveolar mucosa. The sensitivity degree (SE) was monthly evaluated, at the same time point of mobility assessment, during force application, and classified as: 0, when the patient reported no discomfort; 1, when slight discomfort was reported; 2, when bearable pain was reported; and 3, when unbearable pain was reported. Peri-implant biofilm was evaluated with the modified plaque index (MPI) for dental implants. This index uses a score of 0 when there is no detectable plaque; 1 when plaque is recognized onto a probe; 2 when it is visible to the unaided eye; and 3 when there is abundance of soft matter. To verify the technique and/or operator influence, individual associations between techniques with success and failure of these anchorage devices, were evaluated.

## ERROR STUDY

Fourteen lateral cephalograms and CBCTs were randomly selected and remeasured by the same examiner (CCM), after a 30-day interval. Random errors were calculated according to Dahlberg’s formula and systematic errors, with dependent *t*-tests, at *p*<0.05.

## STATISTICAL ANALYSES

Normal distribution was evaluated with Kolmogorov-Smirnov tests. Comparability of the groups regarding sex was evaluated with Chi-square; age and FMA, with *t*-tests. Intergroup cortical bone thickness comparisons and its correlation with FMA were performed with *t*-tests and Pearson’s correlation coefficient, respectively. Intergroup comparisons regarding mobility of the mini-implants and the success rate were performed with Mann-Whitney and Fisher exact tests. Modified plaque index, observation period, and insertion technique/operator were compared between groups with Mann-Whitney and Chi-square tests. To further investigate the factors that could interfere with mini-implants stability, all 56 mini-implants were divided into two groups based on “success” or “failure” condition. Then the following variables were compared between these groups: cortical bone thickness at the insertion site (*t*-test); soft tissue at the insertion site (Chi-square test); modified plaque index, sensitivity during loading, and observation period (Mann-Whitney test); and insertion technique/operator (Fisher’s exact test). All statistical tests were performed with Statistica software (version 7.0, StatSoft Inc., Tulsa, OK, USA). Results were considered significant at *p*<0.05.

## RESULTS

The random errors ranged from 0.07mm (MxLA) to 0.16mm (MdLA) and was 0.57° for the FMA. There were no significant systematic errors. 

The groups were comparable regarding sex ratio and age, but the HG presented a significantly smaller FMA than the VG (Table 2).

**Table 2:** Intergroup comparisons regarding sex, age, FMA, cortical thickness of alveolar bone, mobility, success rate of mini-implants, modified plaque index, observation period and insertion technique/operator variables.

Variables	Horizontal Group (HG)		Vertical Group (VG)		P
	n= 15 patients (26 mini-implants)		n= 15 patients (30 mini-implants)		
Sex	n	%	n	%	
Male	6	40	7	46.67	
Female	9	60	8	53.33	
	Mean	SD	Mean	SD	
Age	25.12	9.48	21.10	8.96	0.24^€^
FMA	19.79	3.61	28.92	3.14	0.00^€^*
Cortical thickness of alveolar bone	Mean	SD	Mean	SD	
Maxillary buccal posterior region (MxBP)	1.19	0.14	1.19	0.23	0.99^€^
Maxillary labial anterior region (MxLA)	1.32	0.13	1.17	0.20	0.02^€^*
Maxillary palatal posterior region (MxPP)	1.59	0.22	1.47	0.29	0.21^€^
Maxillary palatal anterior region (MxPA)	1.47	0.19	1.34	0.36	0.23^€^
Mandibular buccal posterior region (MdBP)	1.73	0.22	1.51	0.26	0.02^€^*
Mandibular labial anterior region (MdLA)	1.24	0.16	1.03	0.20	0.00^€^*
Mandibular lingual posterior region (MdLP)	2.33	0.63	2.28	0.34	0.80^€^
Mandibular lingual anterior region (MdLgA)	2.01	0.54	1.74	0.37	0.12^€^
	Mean	SD	Mean	SD	
Mobility	0.04	0.18	0.09	0.23	0.73^$^
Success rate	n	%	n	%	
Success	24	92.31	26	86.67	0.67^£^
Failure	2	7.69	4	13.33	
	Mean	SD	Mean	SD	
Modified plaque index	1.38	0.75	1.69	0.66	0.11^$^
Observation period	8.35	2.87	9.13	3.46	0.07^$^
Insertion technique/operator	n	%	n	%	
SEB	20	76.92	18	60	0.18^¥^
CCM	6	23.08	12	40	

^¥^Chi-square test, ^€^ t-test, ^$^ Mann-Whitney test, ^£^ Fisher’s exact test. * Statistically significant at p<0.05.

The HG showed significantly greater cortical thickness of alveolar bone at the maxillary labial anterior region, mandibular buccal posterior region and labial anterior region (Table 2).

The cortical thickness of alveolar bone at the maxillary labial anterior region, mandibular labial and lingual anterior regions showed significant negative correlations with the FMA (Table 3).

**Table 3:** Results of the Pearson correlation between the vertical growth pattern (FMA) and thicknesses of the alveolar cortical bone.

	Thickness variables x FMA	Pearson’s correlation coefficient	P
FMA	Maxillary buccal posterior region	-0.16	0.40
	Maxillary labial anterior region	-0.39	0.03*
	Maxillary palatal posterior region	-0.31	0.09
	Maxillary palatal anterior region	-0.17	0.38
	Mandibular buccal posterior region	-0.35	0.06
	Mandibular labial anterior region	-0.49	0.01*
	Mandibular lingual posterior region	-0.02	0.92
	Mandibular lingual anterior region	-0.38	0.04*

*Statistically significant at p<0.05.

Mobility and success rate of mini-implants, modified plaque index, observation period and insertion technique/operator distribution were similar between the horizontal and vertical groups (Table 2).

Failed mini-implants showed significantly greater sensitivity during loading and smaller observation period than succeeded mini-implants (Table 4).

**Table 4:** Analysis of factors associated with mini-implant failures.

Variables	Success		Failure		
	n= 50 mini-implants (89.29%)		n= 6 mini-implants (10.71%)		
Cortical thickness of alveolar bone at the insertion site	Mean	SD	Mean	SD	P
	1.21	0.28	1.17	0.31	0.76^€^
Insertion site soft tissue	n	%	n	%	
Attached gingiva	26	89.66	3	10.34	0.91^¥^
Mucogingival line	13	86.67	2	13.33	
Alveolar mucosa	11	91.67	1	8.33	
	Mean	SD	Mean	SD	
Modified plaque index	1.49	0.71	2.00	0.63	0.17^$^
Sensitivity	0.00	0.00	2.58	0.49	0.00^$^*
Observation period	9.62	2.13	1.67	0.52	0.00^$^*
Technique/operator	n	%	n	%	
SEB	34	89.47	4	10.53	1.00^£^
CCM	16	88.89	2	11.11	

^€^ t-test, ^¥^Chi-square test, ^$^ Mann-Whitney test, ^£^ Fisher’s exact test, *Statistically significant at p<0.05.

## DISCUSSION

Due to controversial reports^1^ ^,^ ^2^ ^,^ ^10^ ^,^ ^12^ on the association between vertical growth pattern and stability of mini-implants, this study intended to clarify these points. It could be argued that the number of evaluated mini-implants was small. However, previous studies evaluating load performance or success rate of mini-implants used similar sample sizes.^20^ ^,^ ^21^ Although sample size calculation for the present study was based only in the assessment of the influence of growth pattern on cortical bone thickness,^13^ the number of mini-implants used to evaluate stability seems reasonable.^13^ ^,^ ^21^

The HG showed greater cortical bone thickness at the maxillary labial anterior region, mandibular buccal posterior and labial anterior regions (Table 2). It could be thought that these results support the concept that subjects with horizontal growth have greater cortical bone thickness.^7^ ^-^ ^11^ ^,^ ^13^ However, this could not be generalized to all regions of the maxilla and the mandible, because some of them did not show significant intergroup differences. Specifically in the region where the MI (posterior maxillary buccal) were installed, there was no difference in cortical thickness, contrary to other studies.^7^ ^,^ ^9^ ^-^ ^11^ In these studies, although there were significant differences between the subjects with horizontal and vertical growth patterns, they were minimal, and could possibly have no clinical significance.

The significant negative correlation found between maxillary and mandibular labial anterior regions with the FMA confirms the results of the intergroup comparisons (Tables 2 and 3). The smaller the FMA, the greater will be the cortical bone thickness at these regions. Nevertheless, the other variable (mandibular buccal posterior region) that showed significant intergroup difference was not significantly correlated with FMA. There was also a significant correlation with the mandibular lingual anterior region (Table 3). Although significant correlations were present, they were not sufficiently strong. Based on these results, we could expect the same success rate of mini-implants in the posterior regions of the maxilla and mandible, independently of the growth pattern. However, the correlations found should be considered when mini-implants are planned to be inserted in the anterior region.

Primary stability of mini-implants is related to thickness of the alveolar cortical bone^4^ ^-^ ^6^ ^,^ ^22^ ^,^ ^23^. Cortical bone thickness should be of at least 1 mm for a mini-implant to be successful^4^. Both groups in this study had thickness greater than 1 mm (Table 2). Thus, it was adequate in both groups, resulting in similar degree of mini-implants mobility (Table 2).

The success rates of the orthodontic mini-implants were not significantly different in patients with HG or VG (Table 2), supporting previous reports^1^. Miyawaki et al.^12^ reported success rates for patients with high mandibular plane angle of 72.7%, and 100% in patients with small mandibular plane angle, which were significantly different. Contrary to the present study, these authors used mini-implants with various diameters, ranging from 1.0 to 2.3 mm and also had several uncontrolled variables that could have influenced their results.^12^ Moon et al.^2^ also examined the relationship between success rate and growth pattern. Even though they suggested that the FMA might be an important factor when success rate is evaluated, no statistically significant differences in the success rate were shown between the low, average and high angle groups. It seems that more important than the growth pattern is the amount of cortical bone thickness.

In the current study, mini-implants were considered successful if did not have any degree of mobility. Several studies suggest that the absence of attached gingiva in the MI insertion site might interfere with its stability.^1^ ^,^ ^24^ ^-^ ^26^ Our results demonstrated that the soft tissue characteristics at the insertion site did not significantly influence the mini-implants stability, as previously reported.^19^

The modified plaque index showed no significant differences between success and failure groups, however, the failure group showed a tendency to have smaller quality of hygiene (Table 4). Studies state the idea that the better the hygiene, the greater the mini-implant success rate.^4^ ^,^ ^12^ ^,^ ^20^ Perhaps our findings were different because the patients were monthly oriented to maintain optimal hygiene in the MI region.

During the monthly mini-implant assessments, it was noticed that the devices without mobility did not have sensitivity, but the degree of sensitivity significantly increased as the mini-implants lost their stability (Table 4). This sensitivity was probably due to compression of the surrounding soft tissues, caused by mini-implants with a high degree of mobility - since, generally, there is no spontaneous pain.^19^ It is reasonable to state that pain sensitivity during mini-implant load is not normal, and this finding could be indicative of mini-implant mobility, resulting in an unfavorable prognosis.^27^ This was the case of the great majority of the failed mini-implants of the present study. When sensitivity is felt during loading, it is suggested to remove the mini-implant, in order to prevent further bone loss and facilitate the reinsertion procedure in an adjacent location.^27^ Mini-implants that failed were evaluated only for 1.67 months, and the successful mini-implants, for 9.62 months. The failed mini-implant period was similar to other studies.^2^ ^,^ ^19^ ^,^ ^24^

In this study, pain sensitivity was evaluated using an ordinal scale. A precise quantification of pain is extremely difficult to obtain and mainly subjective, therefore, it could be argued that a visual analogic scale would be more adequate and practical.^1^ ^,^ ^28^ However, the ordinal scale was also capable to provide reliable results as previously reported.^19^ Both methods of evaluation are consistent.^29^

Various techniques, as well as different operators involved in mini-implant insertion, has been reported in the majority of the studies. However, they did not evaluate the influence of these factors on the stability of orthodontic mini-implants.^1^ ^,^ ^2^ ^,^ ^4^ ^,^ ^12^ ^,^ ^20^ ^,^ ^24^ Kyung et al.^30^ stated that the ability of the operator influences the stability of the MI. However, this can be overridden when the systems are properly calibrated and follow precise insertion techniques,^2^ ^,^ ^16^ ^,^ ^19^ as in the present study. 

Considering the overall results of this study, the growth pattern did not influence the stability or success rate of mini-implants inserted at the maxillary buccal posterior region, specifically. More studies are necessary to evaluate if the growth pattern influences the stability and success rate of mini-implants in other specific and commonly used maxillary and mandibular regions. When planning the use of mini-implants, the specific anatomical region and characteristics of each patient should be always considered.

Even though this study followed a strict methodology, some limitations such as sample size and participation of different operators should be cited. Nonetheless, previous studies^20,21^ presented similar sample sizes, and the operator influences might have been suppressed with proper calibration and precise technique.^2^ ^,^ ^19^ Although both examiners underwent a rigorous clinical calibration before performing the plaque index assessment, reliability of this evaluation was only clinically assessed, and not statistically evaluated.

## CONCLUSIONS

The primary objective of this study was to evaluate the influence of the vertical growth pattern on the alveolar bone cortical thickness and, secondarily, assess the factors related to the stability and success rate of orthodontic mini-implants. 

Based on this specific sample, it can be concluded that growth pattern has an influence on the alveolar bone cortical thickness in specific areas of the maxilla and mandible, but this fact may have no influence in the stability and success rate of mini-implants in the maxillary buccal posterior region. Some specific conclusions:

» Subjects with horizontal growth showed greater cortical thickness of the alveolar bone in some specific regions: at the maxillary labial anterior region, and at mandibular buccal posterior and labial anterior regions.
» There was a negative correlation between the maxillary labial anterior region, and the mandibular labial and lingual anterior regions with the FMA.
» Stability and success rate of mini-implants, placed in the maxillary posterior buccal region, were similar between horizontal and vertical subjects.
» Failed mini-implants showed greater sensitivity during loading when compared to successful mini-implants. Thus, sensitivity during application of force could be an indicative of probable loss of the mini-implant.
